# Morphology-guided dual-level detection and depth localization of cotton terminal buds for precision topping

**DOI:** 10.3389/fpls.2026.1966512

**Published:** 2026-10-14

**Authors:** Chaoqun Wang, Haojun Wen, Zhaohui Zheng, Haixiao Gou, Jie Zhang, Zhuoyan Zhang

**Affiliations:** 1 College of Mechanical and Electrical Engineering, Shihezi University, Shihezi, Xinjiang, China; 2 Key Laboratory of Northwest Agricultural Equipment, Ministry of Agriculture and Rural Affairs, Shihezi, Xinjiang, China

**Keywords:** cotton, machine vision, plant morphology, precision topping, RGB-D localization, small-object detection, terminal bud

## Abstract

Reliable terminal-bud localization is essential for precision cotton topping, yet the small bud core is often obscured by apical leaves and visually confounded with the surrounding canopy. We developed DGQ-YOLO, a lightweight morphology-guided detector that jointly identifies the bud core and a composite terminal-bud region encompassing the core and adjacent apical leaves. This dual-level representation preserves structural cues when the bud core is partly occluded. The model combines C2f_DWR_CA for multiscale, position-sensitive feature extraction, VoVGS-CA for lightweight feature fusion, and QCN loss for quality-aware classification and small-object regression. DGQ-YOLO was evaluated using 2,230 field images of long-staple cotton at the early bud-development stage. It achieved mAP@50 values of 93.2% and 94.1% for the bud core and composite terminal-bud region, respectively. Overall mAP@50 and mAP@50:95 were 93.6% and 81.7%, respectively. Compared with YOLOv8n, DGQ-YOLO improved overall mAP@50 by 2.2 percentage points. Its parameter count decreased from 3.01 to 2.90 million, and its computational cost fell from 8.2 to 7.8 GFLOPs. Combining the centers of the two detected regions with RGB-D depth measurements supported online terminal-bud localization in field experiments. This morphology-informed perception framework supports precision topping under the tested cultivar, developmental stage, and field conditions, although broader agronomic and actuator-level validation is required.

## 1 Introduction

Cotton is a major industrial fiber crop and the primary biological source of textile lint (Zhao and Chen, 2023). Topping suppresses apical dominance, redistributes nutrients, and promotes boll development, with potential benefits for yield and profitability (Zhang et al., 2021). Current approaches are manual, chemical, or mechanical (Liu et al., 2020; Hao et al., 2018; Lu et al., 2018). Manual topping is labor-intensive, whereas chemical topping may pose environmental and crop-safety risks. Mechanical topping offers higher throughput while reducing labor inputs and chemical exposure. As machines become more automated, accurate terminal-bud recognition becomes essential, particularly in densely planted fields (Liu et al., 2021).

Deep-learning methods are increasingly used for cotton terminal-bud detection. However, small buds, complex backgrounds, leaf occlusion, and morphological variation continue to impede accurate, real-time perception. Yasenjiang et al. (2023) simplified Tiny-YOLOv4, added micro CSP-SPP and CARAFE modules, and reduced computation by 33%. Their model achieved an mAP@50 of 82.5% and a precision of 91.5%. Liu (2022) added a decoupled head and an anchor-free strategy to YOLOv4, achieving 90.15% accuracy. Peng et al. (2023) added a small-scale detection layer and CBAM to YOLOv5, obtaining 92.8% bud-core recognition accuracy. Zheng (2022) combined CBAM, ASFF, and GSConv in CSG-YOLOX, which achieved an mAP of 85.81%. Xi et al. (2024) incorporated Hd-ShuffleNetv2, NLAM, BotNet attention, and EIoU loss into YOLOv5s, reaching 91% average detection accuracy. Wu et al. (2026) added CBAM and FSSLoss to YOLOv12s in CFSS-YOLO, which achieved 87.6% precision. Yu et al. (2025) proposed CPD-YOLO for cotton pest and disease detection, integrating UAV and smartphone images with lightweight strategies to enhance multi-scale perception and deployment flexibility.

Despite this progress, field detection remains constrained by bud-core scale, frequent occlusion, and low contrast against the canopy. When the bud core is partly hidden, adjacent apical leaves may still provide structural cues to the composite terminal-bud region. A field-oriented system must therefore integrate morphological representation, small-object detection, computational efficiency, and spatial localization. Luo et al. (2026) developed LMD-Net for rice disease detection in complex field environments, demonstrating the effectiveness of lightweight multi-scale feature extraction and edge-oriented deployment strategies for small targets under background interference.

Here, we present DGQ-YOLO as a morphology-guided technical advance for detecting and localizing cotton terminal buds under field occlusion. The central innovation is a dual-level annotation and perception strategy that treats the small bud core and the composite terminal-bud region formed by the core and adjacent apical leaves as complementary targets. The detector integrates C2f_DWR_CA for multiscale, position-sensitive feature extraction, VoVGS-CA for lightweight feature fusion, and QCN loss for quality-aware classification and small-object regression. We evaluated detection accuracy and computational cost against YOLO-family and cotton-bud baselines, and then coupled both detected regions with RGB-D depth measurements on a mobile field platform. By translating visible cotton-apex morphology into a dual-level localization constraint, the framework connects plant structural information with the spatial perception required for precision topping.

## 2 Materials and methods

### 2.1 Collection of the terminal-bud dataset

Images of cotton terminal buds were collected in loamy fields in Shihezi, Xinjiang, China, from late June to early July 2025. The study used long-staple cotton at the early bud-development stage. Images were acquired using a DJI Osmo Action 3 camera at an image resolution of 640 × 640 pixels. The camera was positioned 25–40 cm above each bud, with the lens approximately perpendicular to the bud. Images were collected under different lighting and background conditions to support model generalization. Sampling covered sunny and cloudy conditions during both mornings and afternoons and combined interval and random sampling approaches. In total, 2,230 raw images were acquired (**Figure 1**). Before augmentation, these images were divided into training, validation, and test sets at a ratio of 7:2:1, corresponding to 1,561, 446, and 223 images, respectively. Partitioning before augmentation prevented augmented variants from crossing dataset splits. Random geometric and illumination perturbations commonly used in agricultural imaging were applied only to the training set, increasing it to 3,712 images.

**Figure 1:**
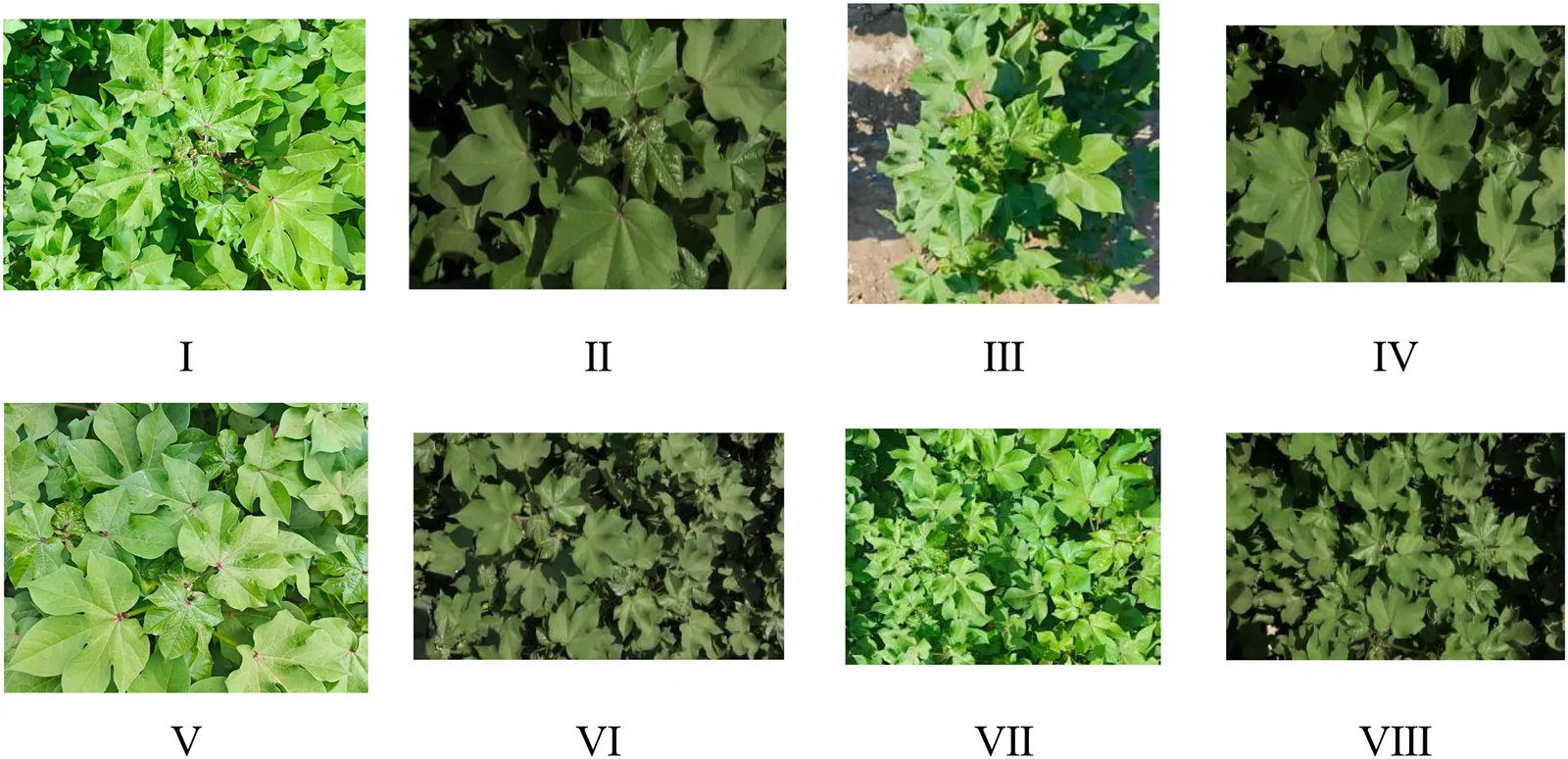
Samples from the cotton terminal bud dataset. (I) Single plant, unobstructed, under strong illumination; (II) single plant, unobstructed, under dim illumination; (III) single plant, occluded under strong illumination; (IV) single plant, occluded under dim illumination; (V) multiple plants, unobstructed, under strong illumination; (VI) multiple plants, unobstructed, under dim illumination; (VII) multiple plants, occluded under strong illumination; (VIII) multiple plants, occluded under dim illumination.

### 2.2 Data annotation

Individual plants in the same field varied in growth vigor, height, leaf expansion, and apical morphology. Previous datasets generally used either a single bud-core box or a two-leaf-one-core annotation scheme. In field conditions, however, the small bud core is often obscured by upper or lateral leaves, particularly in dense stands.

LabelImg was used to annotate the images using a dual-level strategy. The outer blue bounding box denotes the composite terminal-bud region comprising the bud core and adjacent apical leaves, whereas the inner green bounding box denotes the bud core. Thus, only two semantic target classes were used throughout the dataset. The same color convention is applied consistently to the unobstructed, partially occluded, and severely occluded examples shown in **Figure 2**. When the bud core is visible, it can be detected directly. Under partial or severe occlusion, the adjacent apical leaves retain characteristic morphological and spatial cues that constrain the likely location of the bud core.

**Figure 2:**
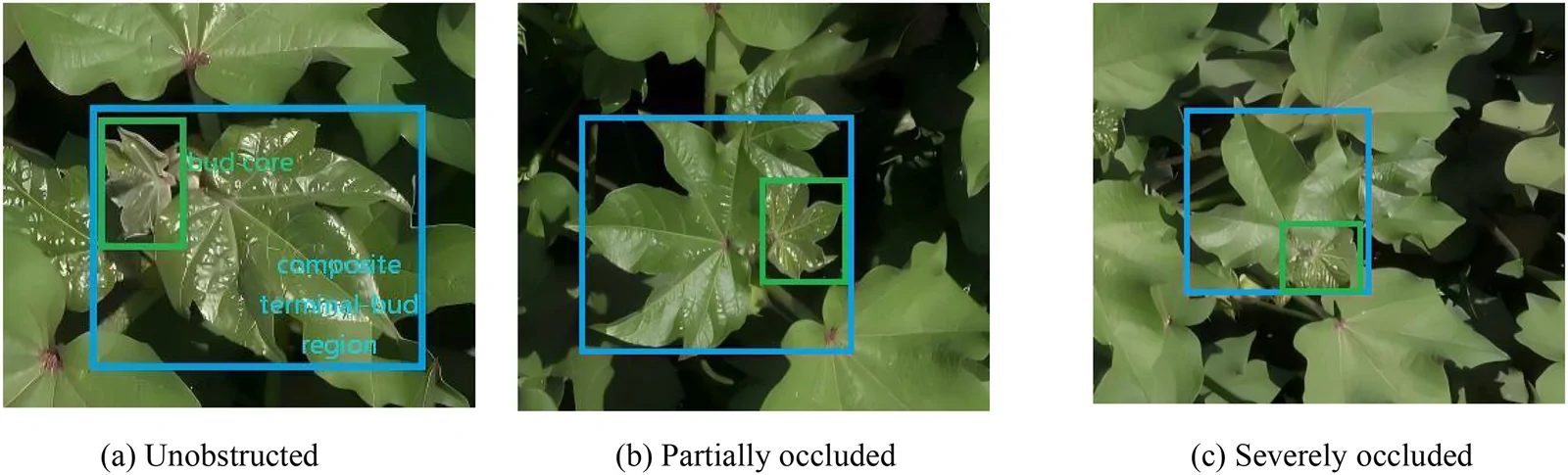
Examples of dual-level annotations of cotton terminal buds under different occlusion conditions: **(a)** unobstructed, **(b)** partially occluded, and **(c)** severely occluded. The blue bounding box denotes the composite terminal-bud region comprising the bud core and adjacent apical leaves, whereas the green bounding box denotes the bud core. The same two annotation classes and color conventions are used in all three examples.

### 2.3 DGQ-YOLO model

DGQ-YOLO is a YOLOv8n-based model for detecting small objects in complex field scenes (**Figure 3**). Its backbone replaces the middle- and high-level C2f modules with C2f_DWR_CA. This module combines C2f_DWR with coordinate attention (CA) (Hou et al., 2021) to capture multiscale context and positional information. In the neck, VoVGS-CA replaces the conventional feature-aggregation module. It uses GSConv within a lightweight fusion structure and applies CA after feature aggregation. The classification branch uses QFL-BCE loss, whereas box regression combines CIoU with NWD for robust small-object localization.

**Figure 3:**
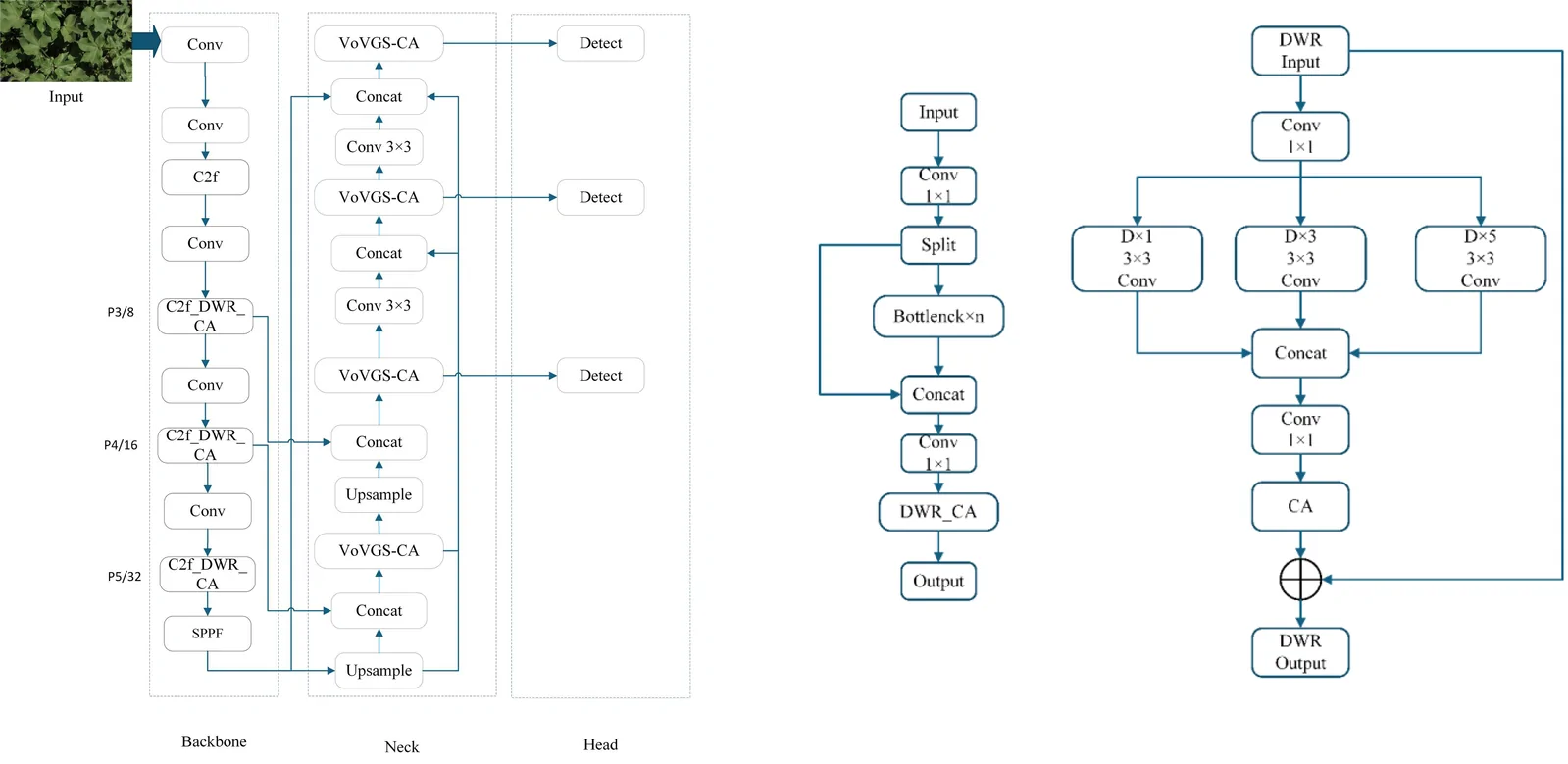
Network structure of DGQ-YOLO, with the detailed structures of the C2f_DWR_CA and DWR_CA modules shown on the right.

### 2.4 C2f_DWR_CA module

C2f_DWR_CA replaces the middle- and high-level C2f modules in the YOLOv8n backbone (**Figure 3**). It retains C2f feature reuse and gradient flow while adding a DWR unit (Wei et al., 2022). DWR was selected to strengthen local texture and multiscale context for small terminal buds against complex backgrounds.

The DWR unit builds multiple receptive fields with low computational overhead. A 1 × 1 convolution first adjusts the input channels. The features then enter three branches containing 3 × 3 convolutions with dilation rates of 1, 3, and 5. Their outputs are concatenated and fused by a 1 × 1 convolution. Coordinate attention weights the fused features along spatial directions, and a residual connection adds them to the input. This design targets local texture, multiscale context, and positional information without a large increase in computation.

### 2.5 VoVGS-CA module

VoVGS-CA was designed to improve multiscale neck fusion while maintaining a lightweight architecture (**Figure 4**). It adopts the one-shot aggregation principle of VoVNet within a dual-branch structure. One branch contains a GSBottleneck built with GSConv (Li et al., 2024), and coordinate attention is applied after branch fusion. Together, lightweight convolution, feature aggregation, and directional attention target weak bud features in complex field scenes.

**Figure 4:**
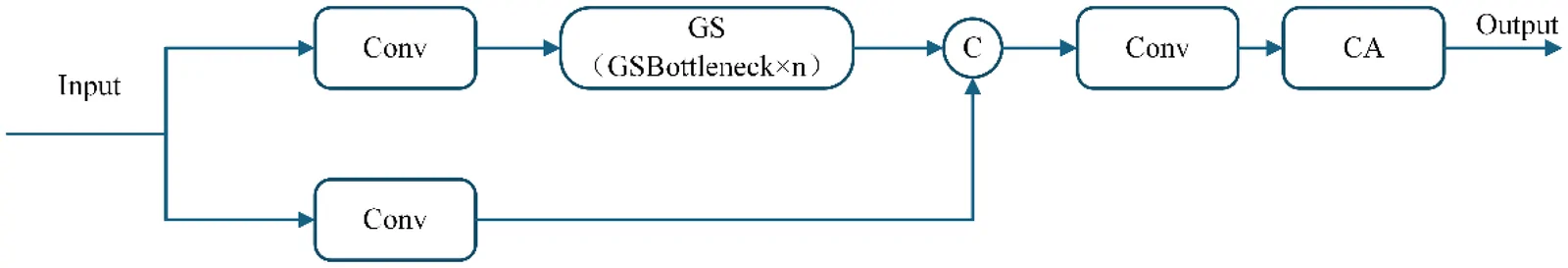
Structure of the VoVGS-CA module.

VoVGS-CA divides the input features into two branches. The first applies a 1 × 1 convolution followed by GSBottleneck for feature extraction. The second applies a 1 × 1 convolution to preserve input detail and provide a direct gradient path. The branch outputs are concatenated, fused by another 1 × 1 convolution, and reweighted by coordinate attention. This architecture combines detail preservation and multiscale fusion at low computational cost.

### 2.6 Loss function optimization

To improve small-object classification and localization, we constructed a task-specific composite objective termed QCN loss. The regression branch combines Complete Intersection over Union (CIoU) and Normalized Wasserstein Distance (NWD) losses while retaining Distribution Focal Loss (DFL). The classification branch combines Quality Focal Loss (QFL) with Binary Cross-Entropy (BCE) and upweights positive samples of the bud-core class.

#### 2.6.1 CIoU-NWD regression loss

For a small terminal bud, even a minor pixel offset between predicted and ground-truth boxes can substantially change the Intersection over Union (IoU). This sensitivity can destabilize regression and reduce localization accuracy. NWD was therefore added to YOLOv8’s CIoU loss as a complementary criterion for position and scale differences. DFL was retained to supervise the discrete distribution of box coordinates and preserve compatibility with the original YOLOv8 representation.

The CIoU loss jointly considers overlap, center-point distance, and aspect-ratio consistency between predicted and ground-truth boxes (Zheng et al., 2022). It is defined as follows (Equations 1–4):

(1) $$L_{CIOU}=1-IoU+\frac{\rho^{2}\left(b,b^{\text{g}t}\right)}{c^{2}}+\alpha v$$

(2) $$\alpha =\frac{v}{\left(1-IoU\right)+v}$$

(3) $$v=\frac{4}{\pi^{2}}{\left(arctan\frac{W_{gt}}{H_{gt}}-arctan\frac{W}{H}\right)}^{2}$$

(4) $$IoU=\left|\frac{A\cap B}{A\cup B}\right|$$

Here, *ρ*^2^(*b*, *b*^gt^) is the squared Euclidean distance between box centers, and *b* and *b*^gt^ are the predicted and ground-truth centers. *c* is the diagonal length of their smallest enclosing rectangle. *α* is the trade-off coefficient, and *v* measures aspect-ratio discrepancy.

NWD quantifies positional and scale differences between predicted and ground-truth boxes. Each box is represented as a two-dimensional Gaussian, and their discrepancy is measured using the Wasserstein distance (Xu et al., 2022). Let the ground-truth and predicted boxes be represented as follows (Equation 5):

(5) $$W_{2}^{2}\left(N_{a},N_{b}\right)={\left||[{\left(cX_{a},cY_{a},\frac{w_{a}}{2},\frac{h_{a}}{2}\right)}^{T}-{\left(cX_{b},cY_{b},\frac{w_{b}}{2},\frac{h_{b}}{2}\right)}^{T}]|\right|}_{2}^{2}$$

Here, *N_a_* and *N_b_* are the Gaussian representations of the ground-truth and predicted boxes, respectively. $A=\left(cX_{a},\;cY_{a},\;w_{a},\;h_{a}\right)$ denotes the ground-truth box, and $B=\left(cX_{b},\;cY_{b},\;w_{b},\;h_{b}\right)$ denotes the predicted box. Because Wasserstein distance is a discrepancy measure, it was converted to a similarity measure by exponential normalization. NWD is calculated as follows (Equation 6):

(6) $$NWD\left(N_{a},N_{b}\right)=exp\left(-\frac{\sqrt{W_{2}^{2}\left(N_{a},N_{b}\right)}}{C}\right)$$

where C is a dataset-dependent normalization constant set to 12.8. The final bounding-box loss is (Equation 7):

(7) $$L_{\text{box}}=\theta L_{\text{CIoU}}+\left(1-\theta \right)L_{NWD}$$

where θ weights the two losses and ranges from 0 to 1. We used equal CIoU and NWD weights (1:1), corresponding to θ = 0.5. Intuitively, CIoU anchors regression to conventional box geometry by enforcing overlap, center-distance, and aspect-ratio consistency, whereas NWD provides a smoother measure of position and scale discrepancy when a few-pixel displacement causes a large IoU change for a small bud. Giving the two terms equal weight prevents the smoother NWD term from weakening geometric box constraints and prevents CIoU from dominating the regression when IoU becomes unstable for tiny targets. Thus, the 1:1 setting provides a balanced compromise between geometric consistency and robustness to small-object positional offsets; this interpretation is also supported empirically by the weight-ratio comparison in **Table 1**.

**Table 1:** Comparison of model performance under different weight ratios for regression loss.

Loss function	Class-wise mAP@50/%		mAP@50%	mAP@50:95%	Parameters (M)	GFLOPs
	Bud core	Composite terminal-bud region				
CIoU:NWD=2:8	92.4	93.7	93	80.6	2.9	7.8
CIoU:NWD=3:7	92.6	93.8	93.2	80.8	2.9	7.8
CIoU:NWD=4:6	92.2	93.2	92.7	80.3	2.9	7.8
CIoU:NWD=5:5	92.8	94	93.4	81.1	2.9	7.8
CIoU:NWD=6:4	92.4	93.3	92.8	80.3	2.9	7.8
CIoU:NWD=7:3	92.6	93.5	93.0	80.5	2.9	7.8

#### 2.6.2 QFL-BCE classification loss

YOLOv8 uses Binary Cross-Entropy (BCE) as the basic classification objective. Although BCE provides stable optimization, field images contain many background samples and small, frequently occluded bud cores. Background samples may therefore dominate training and weaken discrimination of the target classes.

We combined QFL and BCE and assigned greater weight to positive samples of the bud-core class. This design was intended to increase sensitivity to the primary target while retaining BCE’s optimization stability.

The BCE loss is defined as follows (Equation 8):

(8) $$L_{BCE}=-\frac{1}{N}\overset{N}{\underset{i=1}{\sum }}\left[y_{i}\log\left(p_{i}\right)+\left(1-y_{i}\right)\log\left(1-p_{i}\right)\right]$$

where $y_{i}\in \left\{0,1\right\}$ is the ground-truth binary label and *p_i_* is the predicted probability of the positive class. BCE measures the discrepancy between predicted probabilities and binary labels, with smaller values indicating closer agreement.

Quality Focal Loss (QFL) was added to align classification confidence with localization quality. Unlike losses based only on discrete class labels, QFL includes target quality in classification supervision. The resulting score therefore reflects both class confidence and box quality, which can help distinguish small, difficult samples (Li et al., 2020). QFL is defined as follows (Equation 9):

(9) $$L_{QFL}=-{\left|d-p_{i}\right|}^{\beta }\left(1-d\right)\log\left(1-p_{i}\right)+d\log\left(p_{i}\right)$$

where *d* is the target quality score in [0,1], *p_i_* is the predicted score, and *β* is the modulation exponent. We set β to 1.5 to control the relative influence of easy and difficult samples.

To retain BCE stability while adding quality-aware supervision, QFL and BCE were equally weighted (Equation 10):

(10) $$L_{cls}=\omega_{i}\left(0.5L_{QFL}+0.5L_{BCE}\right)$$

where ω_i_ is the class-priority weight. Positive bud-core samples were assigned a weight of 1.3, and all other samples a weight of 1. This weighting emphasizes the primary class without changing the network architecture.

The final detection objective combines bounding-box regression, classification, and Distribution Focal Loss (Equation 11):

(11) $$L=L_{box}+L_{cls}+L_{DFL}$$

where *L_box_*, *L_cls_*, and *L_DFL_* denote the CIoU-NWD regression, QFL-BCE classification, and Distribution Focal losses, respectively. Together, these components constitute QCN loss. The objective jointly targets classification quality and robust localization of small cotton terminal buds.

### 2.7 Experimental platform and evaluation metrics

Experiments were conducted on 64-bit Windows 11 using an Intel Core i5-13650HX processor and a GPU with 8 GB VRAM. The model was implemented using Ultralytics YOLO v8.2.0 with PyTorch 2.0.1 and constructed from a customized YOLOv8n configuration file. No external pretrained weights were explicitly loaded during training. A fixed random seed of 0 was used for dataset loading and model training to improve the reproducibility of the experiments. Models were trained for 200 epochs with a batch size of 8 and four data-loading workers. Stochastic gradient descent (SGD) was used as the optimizer, with an initial learning rate of 0.008, momentum of 0.937, and weight decay of 5 × 10^-4^. The learning rate was adjusted using a cosine decay schedule over the 200 training epochs, with the final learning-rate factor (lrf) set to 0.01. A five-epoch warm-up strategy was adopted, with an initial warm-up momentum of 0.8 and a warm-up bias learning rate of 0.1. The input image size was set to 640 × 640 pixels during training. Data augmentation included HSV perturbation, translation, scaling, horizontal flipping, Mosaic, and MixUp. The probabilities of Mosaic and MixUp were set to 0.8 and 0.1, respectively, and Mosaic augmentation was disabled during the final 10 epochs to improve bounding-box localization. Automatic mixed-precision (AMP) training was enabled, and a patience value of 80 epochs was used for early stopping.

Performance was evaluated using mean average precision (mAP), parameter count, and giga floating-point operations (GFLOPs). Because the intended application involves field deployment, we emphasized the balance between detection accuracy and computational cost. These metrics provide a basis for assessing the model’s suitability for subsequent real-time perception and robotic cotton topping.

## 3 Results

### 3.1 Analysis of backbone improvements

Comparative experiments evaluated the backbone modification and several attention mechanisms. Adding C2f_DWR increased mAP@50 from 90.8% to 91.9% for the bud core and from 92.1% to 93.1% for the composite terminal-bud region (**Table 2**). Overall mAP@50 rose from 91.4% to 92.5%, while parameters and cost increased from 3.01 M and 8.2 GFLOPs to 3.15 M and 8.4 GFLOPs. Thus, C2f_DWR improved both class-specific metrics with a limited increase in model complexity.

**Table 2:** Detection performance of different backbone improvement schemes.

Module	Class-wise mAP@ 50/%		mAP@50/%	mAP@50:95%	Parameters (M)	GFLOPs
	Bud core	Composite terminal-bud region				
YOLOv8n	90.8	92.1	91.4	79.1	3.01	8.2
+C2f_DWR	91.9	93.1	92.5	80.1	3.15	8.4
+C2f_DWR_GAM	91.7	92.5	92.1	79.9	5.6	12.3
+C2f_DWR_NAM	91.5	93.0	92.2	80.0	3.16	8.4
+C2f_DWR_CBAM	92.0	93.1	92.5	80.2	3.16	8.4
+C2f_DWR_SE	91.8	93.0	92.4	80.1	3.16	8.4
+C2f_DWR_CA	92.1	93.4	92.7	80.3	3.16	8.4

Combining C2f_DWR with coordinate attention (CA) (Hou et al., 2021) produced the highest overall performance among the tested attention mechanisms. mAP@50 reached 92.1% for the bud core and 93.4% for the composite terminal-bud region; overall mAP@50 was 92.7%. Parameters and computational cost were 3.16 M and 8.4 GFLOPs. CBAM (Woo et al., 2018) and SE (Hu et al., 2018) also improved the baseline but remained below CA in overall mAP@50. NAM (Liu et al., 2021b) and GAM (Liu et al., 2021a) provided smaller gains, while GAM substantially increased parameters and GFLOPs. C2f_DWR_CA was therefore selected for the final backbone because it provided the strongest accuracy-complexity balance in this comparison.

### 3.2 Performance of lightweight neck modules

The neck was redesigned using ShuffleNetV2 (Gao et al., 2025), MobileNetV3 (Howard et al., 2019), DSConv (Yang et al., 2024), or GSConv (Li et al., 2024). Each variant exceeded baseline YOLOv8n in detection performance while reducing parameters and GFLOPs (**Table 3**). These comparisons show that lightweight convolutions can reduce neck complexity without necessarily sacrificing feature-fusion performance.

**Table 3:** Effects of different lightweight convolutional modules on neck feature fusion.

Module	Class-wise mAP@50/%		mAP@50%	mAP@50:95%	Parameters (M)	GFLOPs
	Bud core	Composite terminal-bud region				
YOLOv8n	90.8	92.1	91.4	79.1	3.01	8.2
+ShuffleNetV2	91.9	93.0	92.4	80.1	2.2	6.4
+MobileNetV3	92.1	92.8	92.5	80.2	2.6	7.3
+DSConv	92.3	93.3	92.8	80.4	2.4	7.0
+GSConv	92.5	93.4	92.9	80.5	2.3	6.6

ShuffleNetV2 produced the largest complexity reduction but only a modest mAP@50 improvement. MobileNetV3 provided a more balanced result, whereas DSConv and GSConv achieved higher detection metrics. With GSConv, mAP@50 reached 92.5% for the bud core and 93.4% for the composite terminal-bud region; overall mAP@50 was 92.9%. It required 2.30 M parameters and 6.6 GFLOPs, giving the best accuracy-cost balance among the tested neck modules.

### 3.3 Comparison of loss functions

We first compared CIoU: NWD weight ratios to address localization errors in small terminal buds. Equal weighting (5:5) produced the best tested performance (**Table 1**). mAP@50 reached 92.8% for the bud core and 94.0% for the composite terminal-bud region; overall mAP@50 was 93.4%. Higher NWD weights weakened geometric box constraints, whereas higher CIoU weights reduced the complementary contribution of NWD. Equal weighting therefore provided the best observed balance between geometric constraints and small-object offset robustness.

We next varied the positive-sample weight for the bud-core class. Performance was highest at a weight of 1.3 (**Table 4**). mAP@50 reached 93.2% for the bud core and 94.1% for the composite terminal-bud region; overall mAP@50 was 93.6%. Larger weights reduced overall performance, indicating that excessive emphasis on the bud-core class disrupted class balance. We therefore selected ωi=1.3.

**Table 4:** Model performance under different positive sample weights of bud core class.

Bud core positive sample weight	Class-wise mAP@50/%		mAP@50%	mAP@50:95%	Parameters (M)	GFLOPs
	Bud core	Composite terminal-bud region				
ω_i_ = 1.3	93.2	94.1	93.6	81.7	2.9	7.8
ω_i_ = 1.5	92.9	93.6	93.2	80.8	2.9	7.8
ω_i_ = 1.8	92.8	93.9	93.3	80.2	2.9	7.8
ω_i_ = 2.0	92.6	93.5	93.1	80.7	2.9	7.8

We then compared regression, classification, and combined losses under matched experimental conditions. Regression losses comprised CIoU, SIoU (Gevorgyan, 2022), EIoU (Zhang et al., 2022), NWD (Xu et al., 2022), and CIoU-NWD; classification losses comprised Focal Loss (Lin et al., 2017) and QFL (Li et al., 2020). QCN was evaluated as the combined objective. Training curves showed that all models approached convergence by 200 epochs (**Figure 5**). QCN maintained the highest and most stable late-stage mAP@50 among the compared objectives.

**Figure 5:**
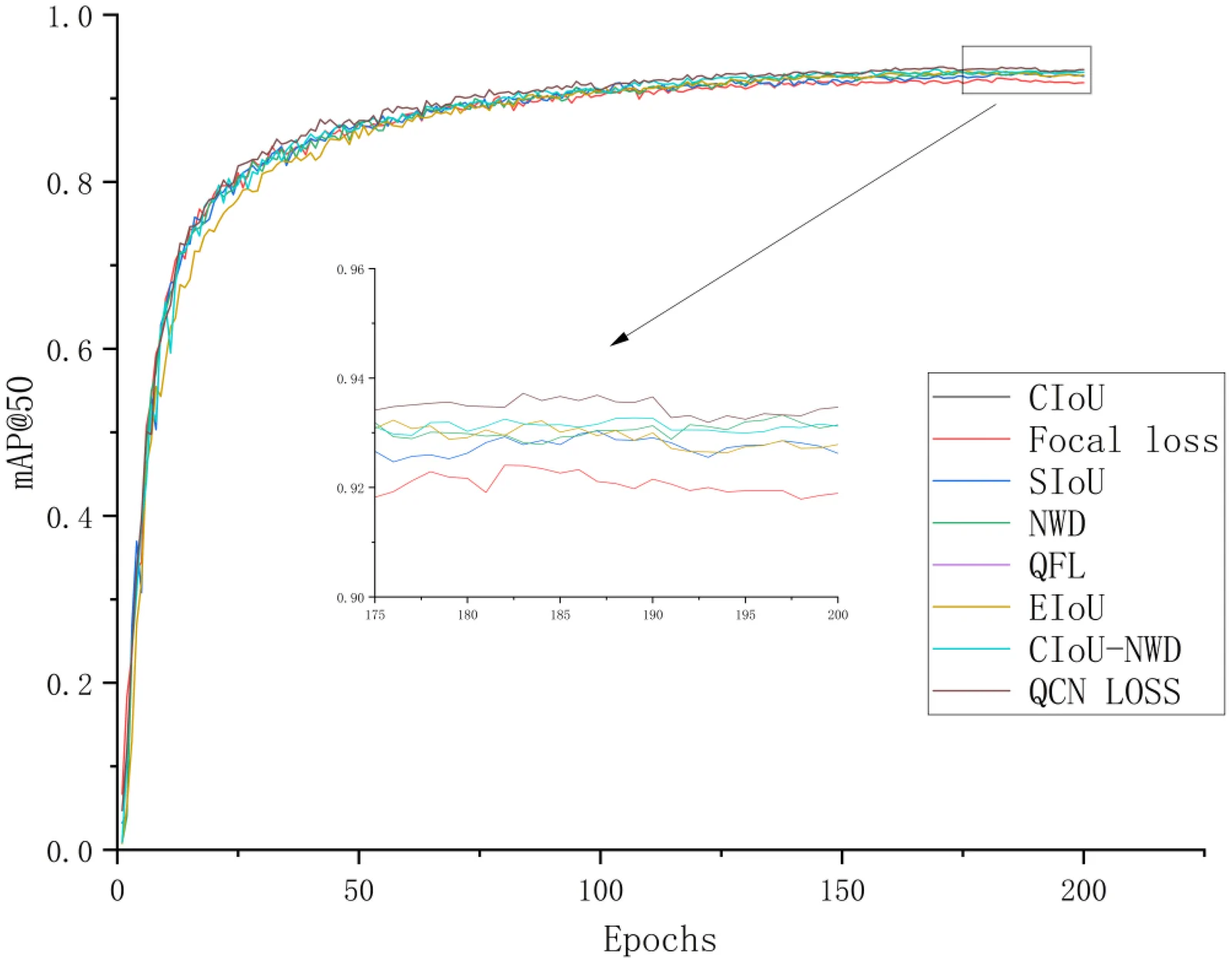
Curve of mAP@50 variation during training with different loss functions.

SIoU and EIoU produced overall mAP@50 values of 92.8% and 92.5%, neither exceeding the CIoU baseline (**Table 5**). NWD reached 93.2%, compared with 92.8% for CIoU. Combining CIoU and NWD further increased overall mAP@50 to 93.4%. Class-specific mAP@50 was 92.8% for the bud core and 94.0% for the composite terminal-bud region. These results support combining box-geometry constraints with distribution-based modeling of small-object offsets.

**Table 5:** Comparison of different loss functions.

Loss function	Class-wise mAP@50/%		mAP@50%	mAP@50:95%	Parameters(M)	GFLOPs
	Bud core	Composite terminal-bud region				
CIoU	92.5	93	92.8	80.5	2.9	7.8
SIoU	92.2	93.4	92.8	80.3	2.9	7.8
EIoU	91.7	93.2	92.5	80.1	2.9	7.8
NWD	92.6	93.9	93.2	81.0	2.9	7.8
Focal loss	91.2	92.1	91.7	79.6	2.9	7.8
QFL	92.5	93.3	92.9	80.4	2.9	7.8
CIoU-NWD	92.8	94.0	93.4	81.1	2.9	7.8
QCN loss	93.2	94.1	93.6	81.7	2.9	7.8

For classification, Focal Loss produced an overall mAP@50 of 91.7%, compared with 92.9% for QFL. This difference supports quality-aware classification for small, difficult targets in complex backgrounds. QCN produced the highest tested mAP@50: 93.2% for the bud core, 94.1% for the composite terminal-bud region, and 93.6% overall.

### 3.4 Ablation study

Ablation experiments evaluated each modification on the same dataset (**Table 6**). YOLOv8n served as the baseline. Model A added C2f_DWR, and Model B replaced it with C2f_DWR_CA. Model C added VoV-GS to Model B, whereas Model D added VoVGS-CA. Model E combined Model D with QCN loss, and Model F combined Model C with QCN loss.

**Table 6:** Results of the ablation experiments.

Model	C2f_DWR	C2f_DWR _CA	VoV-GS	VoVGS- CA	QCN loss	Class-wise mAP@50/%		mAP@50%	Parameters (M)	GFLOPs
						Bud core	Composite terminal-bud region			
Baseline						90.8	92.1	91.4	3.0	8.2
A	✓					91.9	93.1	92.5	3.15	8.4
B		✓				92.1	93.4	92.7	3.16	8.4
C		✓	✓			92.2	93.6	92.9	2.9	7.8
D		✓		✓		91.7	93.2	92.5	2.9	7.8
E		✓		✓	✓	93.2	94.1	93.6	2.9	7.8
F		✓	✓		✓	92.8	94.0	93.4	2.9	7.8

Baseline YOLOv8n achieved an overall mAP@50 of 91.4% (**Table 6**). Introducing C2f_DWR increased it to 92.5%, indicating that multiscale dilated convolutions improved the extraction of small bud features. Adding coordinate attention further increased mAP@50 to 92.7% with nearly unchanged computational cost, suggesting that positional attention complements multiscale feature extraction and improves focus on terminal-bud regions.

Replacing the neck with VoV-GS increased mAP@50 to 92.9% while reducing parameters from 3.16 M to 2.90 M and GFLOPs from 8.4 to 7.8, demonstrating an improved accuracy–complexity balance. However, VoVGS-CA alone achieved 92.5%, indicating that the additional attention mechanism did not independently improve accuracy. Its contribution was more evident when combined with the optimized loss.

Adding QCN loss to VoVGS-CA increased bud-core, composite-region, and overall mAP@50 from 91.7%, 93.2%, and 92.5% to 93.2%, 94.1%, and 93.6%, respectively. Similar improvements were observed when QCN was combined with VoV-GS. These results indicate that CIoU-NWD improves robustness to small-box localization offsets, while QFL-BCE enhances quality-aware classification of difficult bud-core samples. Overall, C2f_DWR_CA strengthens multiscale and positional feature extraction, VoVGS-CA provides lightweight cross-scale fusion, and QCN further improves small-object optimization, resulting in the best overall performance of Model E.

### 3.5 Comparison with other models

For the comparisons reported in **Table 7**, all models, including YOLOv5n, YOLOv5s, YOLOv8n, YOLOv8s, YOLOv11n, YOLOv11s, Improved YOLOv5s, YOLOv5-cpp, Bud-YOLO, and DGQ-YOLO, were retrained under identical experimental conditions. Therefore, the performance differences reported in **Table 7** primarily reflect differences in model architecture rather than variations in training or evaluation conditions.

**Table 7:** Detection results of different detection networks.

Model	Class-wise mAP@50/%		mAP@50%	mAP@50:95%	Parameters (M)	GFLOPs
	Bud core	Composite terminal-bud region				
YOLOv5n	89.3	92.5	90.9	78.6	1.77	4.2
YOLOv5s	91.2	92.6	91.9	79.5	7.0	16.0
YOLOv8s	92.4	93	92.7	80.6	11.1	28.6
YOLOv8n	90.8	92.1	91.4	79.1	3.01	8.2
YOLOv11s	92.0	93.6	92.8	80.7	9.43	21.6
YOLOv11n	90.6	91.7	91.1	79.0	2.59	6.4
Improved YOLOv5s	91.7	92.8	92.2	80.2	3.0	7.3
YOLOv5-cpp	92.6	93.8	93.2	81.0	2.4	6.3
Bud-YOLO	92.9	93.4	93.1	80.9	3.9	9.0
DGQ-YOLO	93.2	94.1	93.6	81.7	2.9	7.8

Relative to YOLOv8n, DGQ-YOLO increased mAP@50 from 90.8% to 93.2% for the bud core and from 92.1% to 94.1% for the composite terminal-bud region. Overall mAP@50 increased from 91.4% to 93.6%. Parameters decreased from 3.01 M to 2.90 M, and computational cost fell from 8.2 to 7.8 GFLOPs.

DGQ-YOLO also exceeded the larger YOLOv8s and YOLOv11s models in overall detection accuracy while using fewer resources. Relative to YOLOv8s, it reduced parameters by 73.9% and GFLOPs by 72.7%. Relative to YOLOv11s, the reductions were 69.2% and 63.9%, respectively. Its overall mAP@50 also exceeded improved YOLOv5s (Xi et al., 2024), YOLOv5-cpp (Peng et al., 2023), and Bud-YOLO (Zhang and Chen, 2024) in this comparison. DGQ-YOLO therefore provided the best observed balance among accuracy, parameters, and computational cost.

Three test-set field images were randomly selected for qualitative comparison (**Figure 6**). YOLOv5n and YOLOv11n each missed two terminal buds, whereas YOLOv8n missed one; red circles indicate representative missed detections. No corresponding missed detections were observed for DGQ-YOLO in these selected examples. The comparison provides qualitative support for improved small-object detection.

**Figure 6:**
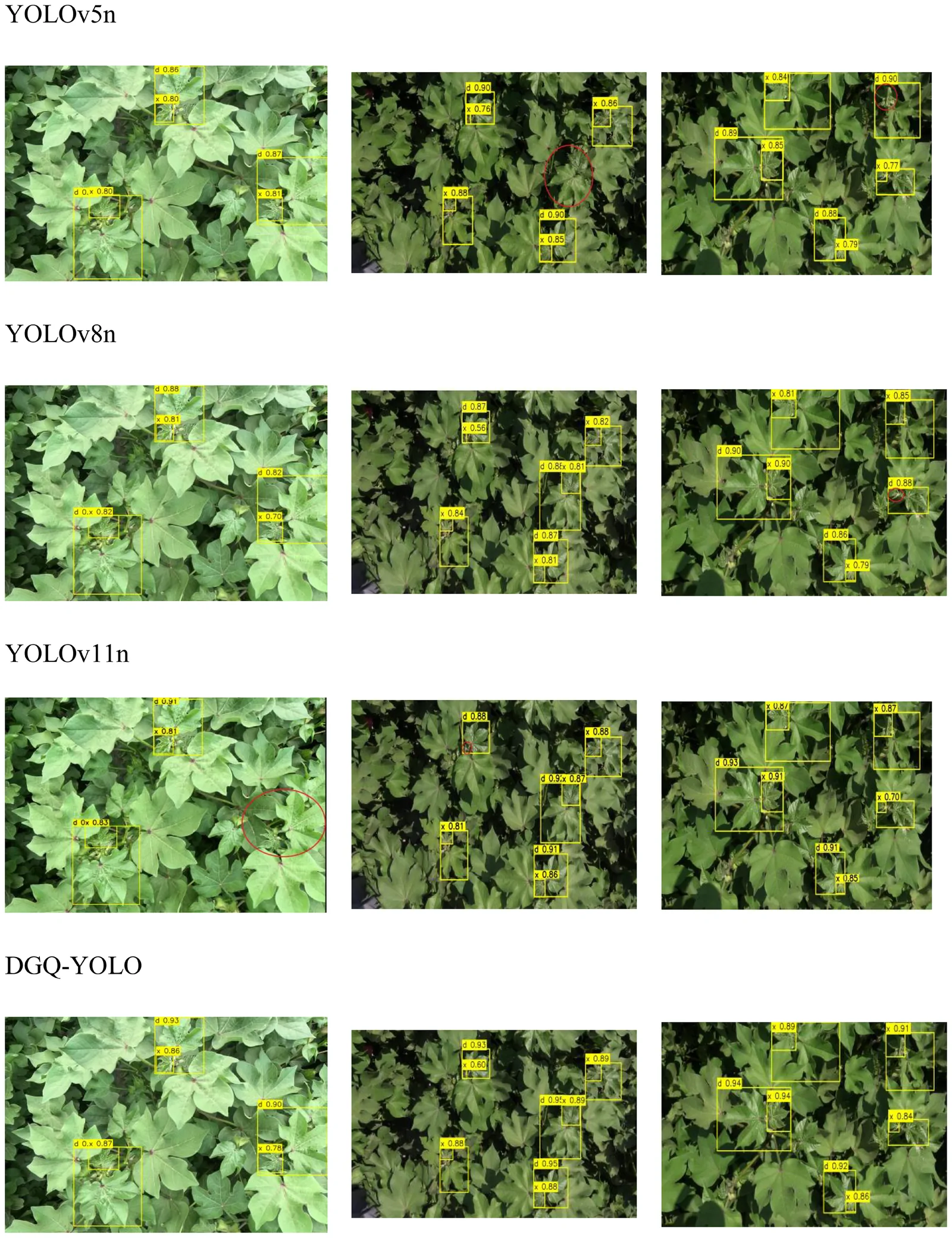
Comparison of detection results obtained by different models.

Before module modification, attention maps were dispersed and included substantial background activation (**Figure 7**). After modification, responses were more concentrated on the bud core and apical leaves. This pattern was consistently observed across the three representative field scenes shown in **Figure 7**. The visualization therefore suggests that DGQ-YOLO suppressed irrelevant background responses and focused more consistently on the annotated regions.

**Figure 7:**
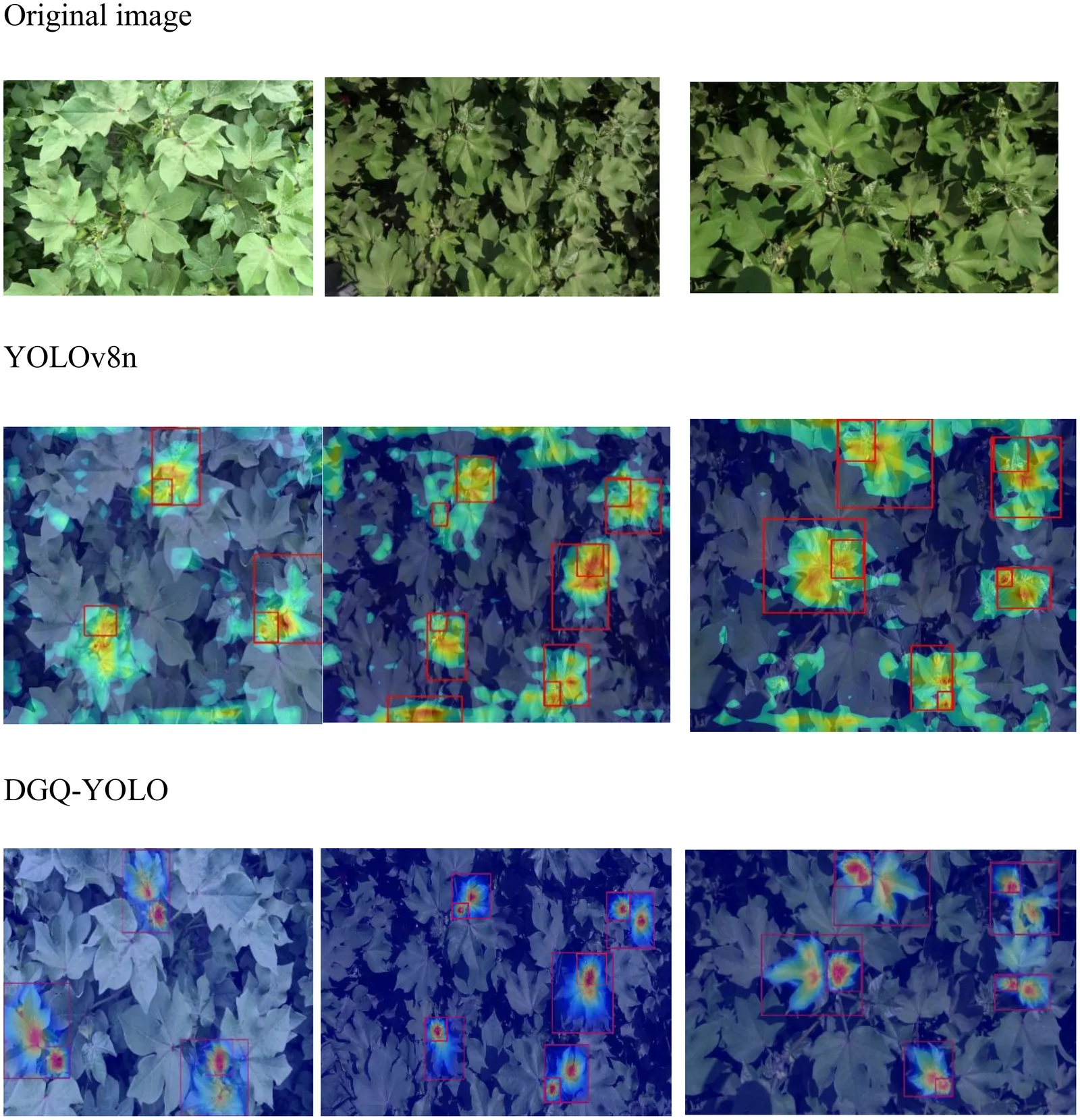
Visualization of attention maps for cotton terminal-bud detection.

### 3.6 Field experiment

An online field platform was developed to evaluate the field applicability of DGQ-YOLO under natural cotton-growing conditions (**Figure 8**). The system consisted of an Intel RealSense D455 RGB-D camera, a laptop, and a mobile field platform. Both RGB and depth images were acquired at a resolution of 1280 × 720 pixels and a frame rate of 30 fps. The camera was mounted at the front of the platform with its optical axis oriented toward the cotton canopy. Prior to depth extraction, the depth images were spatially aligned with the corresponding RGB images to ensure pixel-level correspondence between the detected terminal-bud location and its depth information.

**Figure 8:**
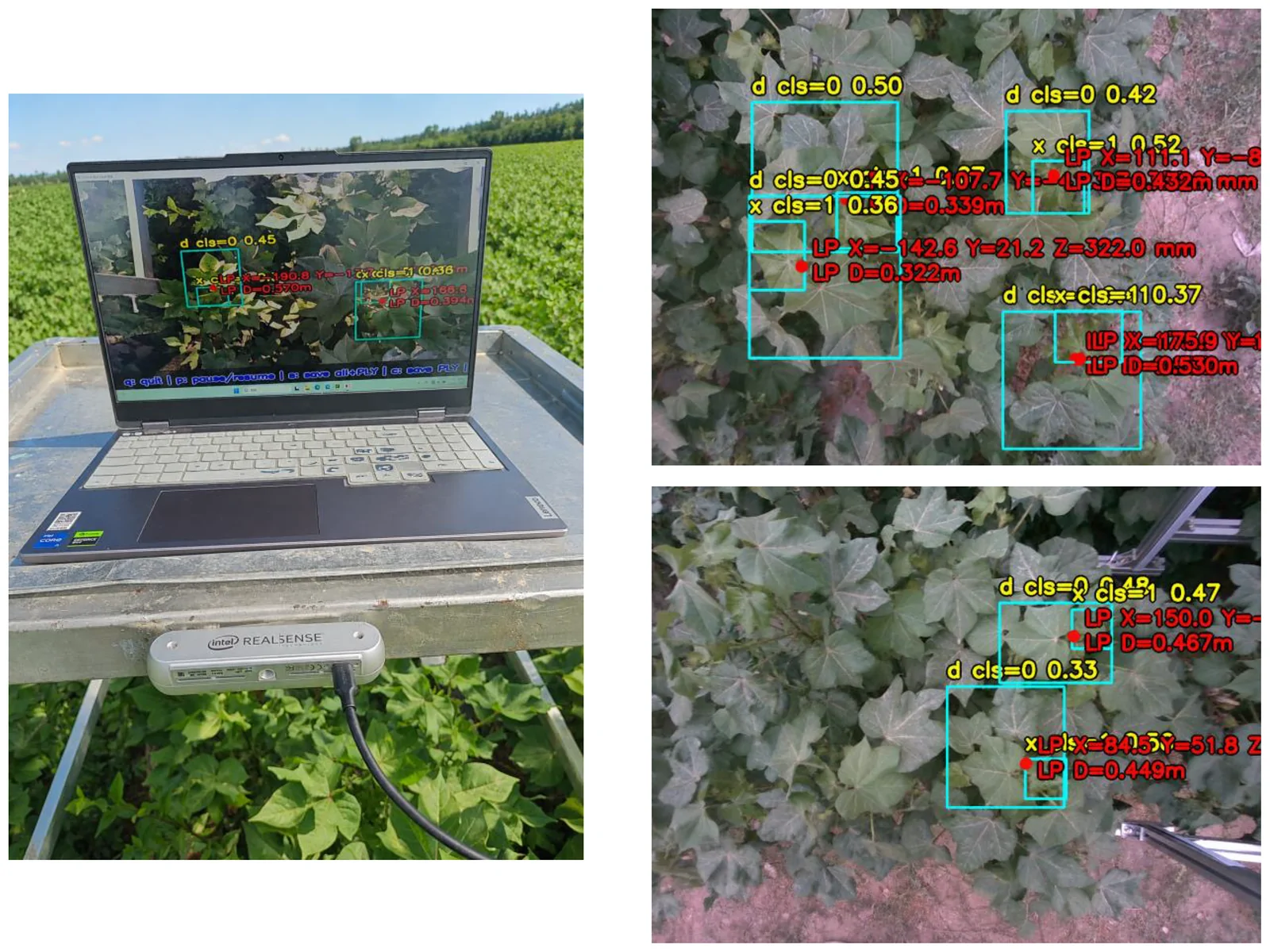
Field-based online detection and localization platform for cotton terminal buds using the midpoint of dual bounding boxes.

Three-dimensional localization was performed in the RGB camera coordinate system after depth-to-color alignment. The origin was defined at the optical center of the RGB camera, with the +X axis directed to the right of the image, the +Y axis directed downward, and the +Z axis extending forward along the optical axis. After detecting the bud-core and composite terminal-bud regions, the midpoint of the two bounding-box centers was calculated as the terminal-bud localization point. The corresponding aligned depth value was then extracted, and the pixel coordinates together with the depth measurement were deprojected into three-dimensional coordinates (X, Y, Z) using the calibrated intrinsic parameters of the camera. This coordinate system was used consistently for the measured coordinates and localization errors reported in **Table 8**.

**Table 8:** Three-dimensional coordinate measurements and localization errors.

No.	Measured value (mm)	Reference value (mm)	Error (mm)
1	(84.5,51.8,449)	(83,53,446)	(1.5,−1.2,3)
2	(150,-121.3,467)	(148,-123,464)	(2,1.7,3)
3	(-142.6,21.2,322)	(-140,23,323)	(−2.6,−1.8,−1)
4	(175.9,195.3,530)	(173,197,534)	(2.9,−1.7,−4)
5	(111.1,-83.7,432)	(109,-85,435)	(2.1,1.3,−3)
6	(116.4,29.6,351)	(113,25,349)	(3.4,4.6,2)
7	(-27.3,29.4,296)	(-24,32,293)	(−3.3,−2.6,3)
8	(63.7,36.5,394)	(61,39,392)	(2.7,−2.5,2)
9	(40.9,86.3,356)	(38,84,352)	(2.9,2.3,4)
10	(9.7,43,267)	(7,41,263)	(2.7,2,4)

For quantitative localization-accuracy evaluation, the mobile platform was kept stationary during coordinate measurement to minimize motion-induced uncertainty. Ground-truth coordinates were manually obtained using a measuring tape with reference to the RGB camera coordinate origin. The distances from each selected terminal-bud localization point to the reference origin along the X, Y, and Z directions were measured repeatedly to reduce random manual measurement error, and the resulting mean values were used as the ground-truth coordinates. For each test point, the three-dimensional coordinates estimated by the RGB-D localization system were compared with the corresponding ground-truth coordinates. The localization errors along the X, Y, and Z axes were calculated as $\Delta X=X_{est}-X_{ref}$, $\Delta Y=Y_{est}-Y_{ref}$, and $\Delta Z=Z_{est}-Z_{ref}$, respectively. The quantitative localization results are summarized in **Table 8**. In addition, a separate moving-platform trial was conducted under natural illumination as a qualitative demonstration of the online applicability of the proposed detection and localization framework under practical field operating conditions.

The inner box represented the bud core, whereas the outer box represented the composite terminal-bud region. When the core was visible, it provided fine-grained positional information. Under partial occlusion, the adjacent leaves preserved regional cues through their morphology and spatial arrangement.

During localization, the centers of the inner and outer boxes were calculated separately. Let their coordinates be $C_{i}\left(x_{i},y_{i}\right)$ and $C_{o}\left(x_{o},y_{o}\right)$, respectively. The terminal-bud localization point was defined as their midpoint, $LP\left(\frac{x_{i}+x_{o}}{2},\frac{y_{i}+y_{o}}{2}\right)$. This calculation combines precise bud-core information with the regional constraint of the composite box. It may therefore reduce sensitivity to occlusion, small target size, and box displacement.

Both boxes were detected simultaneously, and their midpoint was calculated online (**Figure 8**). The point lay within the composite terminal-bud region, and the RGB-D camera provided the corresponding depth. The localization results are summarized (**Table 8**).

The field experiment showed that the dual-level representation remained detectable under occlusion and supported a stable geometric localization point. These online results provide preliminary evidence for midpoint localization using boxes for the bud core and composite terminal-bud region. They establish a basis for subsequent three-dimensional localization and actuator-level testing in robotic cotton topping.

## 4 Conclusion

This study presents DGQ-YOLO, a morphology-guided, dual-level framework for detecting and localizing cotton terminal buds in complex field scenes. By jointly representing the bud core and a composite terminal-bud region that incorporates adjacent apical leaves, the framework retains complementary structural cues when the core is partly occluded. C2f_DWR_CA, VoVGS-CA, and QCN loss together enhance multiscale feature extraction, lightweight fusion, quality-aware classification, and small-object regression.

Under the study’s evaluation conditions, DGQ-YOLO achieved mAP@50 values of 93.2% and 94.1% for the bud core and composite terminal-bud region, respectively. Overall mAP@50 and mAP@50:95 were 93.6% and 81.7%, respectively. Relative to YOLOv8n, DGQ-YOLO improved overall mAP@50 by 2.2 percentage points while reducing its parameter count and computational cost. Field experiments further indicated that the midpoint of the two detected regions, combined with RGB-D depth measurements, can support online terminal-bud localization. These results provide a technical basis for precision cotton topping, but the evidence is limited to long-staple cotton at the early bud-development stage in one location. Validation across cultivars, growth stages, locations, seasons, occlusion levels, embedded hardware, and actuator-controlled topping is required before operational deployment.

## Data Availability

The raw data supporting the conclusions of this article will be made available by the authors, without undue reservation.
